# A causal inference study: The impact of the combined administration of Donepezil and Memantine on decreasing hospital and emergency department visits of Alzheimer’s disease patients

**DOI:** 10.1371/journal.pone.0291362

**Published:** 2023-09-14

**Authors:** Ehsan Yaghmaei, Albert Pierce, Hongxia Lu, Yesha M. Patel, Louis Ehwerhemuepha, Ahmad Rezaie, Seyed Ahmad Sajjadi, Cyril Rakovski

**Affiliations:** 1 Schmid College of Science and Technology, Chapman University, Orange, CA, United States of America; 2 School of Medicine, University of North Carolina at Chapel Hill, Chapel Hill, NC, United States of America; 3 Children’s Hospital of Orange County (CHOC), Orange, CA, United States of America; 4 School of Medicine, University of California, Irvine, Irvine, CA, United States of America; National Center of Neurology and Psychiatry (NCNP), JAPAN

## Abstract

Alzheimer’s disease is the most common type of dementia that currently affects over 6.5 million people in the U.S. Currently there is no cure and the existing drug therapies attempt to delay the mental decline and improve cognitive abilities. Two of the most commonly prescribed such drugs are Donepezil and Memantine. We formally tested and confirmed the presence of a beneficial drug-drug interaction of Donepezil and Memantine using a causal inference analysis. We applied doubly robust estimators to one of the largest and high-quality medical databases to estimate the effect of two commonly prescribed Alzheimer’s disease (AD) medications, Donepezil and Memantine, on the average number of hospital or emergency department visits per year among patients diagnosed with AD. Our results show that, compared to the absence of medication scenario, the Memantine monotherapy, and the Donepezil monotherapy, the combined use of Donepezil and Memantine treatment significantly reduces the average number of hospital or emergency department visits per year by 0.078 (13.8%), 0.144 (25.5%), and 0.132 days (23.4%), respectively. The assessed decline in the average number of hospital or emergency department visits per year is consequently associated with a substantial reduction in medical costs. As of 2022, according to the Alzheimer’s Disease Association, there were over 6.5 million individuals aged 65 and older living with AD in the US alone. If patients who are currently on no drug treatment or using either Donepezil or Memantine alone were switched to the combined used of Donepezil and Memantine therapy, the average number of hospital or emergency department visits could decrease by over 613 thousand visits per year. This, in turn, would lead to a remarkable reduction in medical expenses associated with hospitalization of AD patients in the US, totaling over 940 million dollars per year.

## 1. Introduction

Alzheimer’s disease (AD) is the most common form of dementia and may contribute to 60–70% of all cases [1, 2]. Globally, more than 50 million individuals are affected by dementia, and projections indicate that this number will increase by over threefold by 2050 due to population aging assuming no medical breakthroughs to prevent, slow or cure AD are made. Currently, AD is listed as one of the top five causes of mortality worldwide and is the seventh leading cause of death in the United States [1, 2]. Per the Alzheimer’s Disease Association’s 2022 report, the prevalence of AD in the United States has now surpassed 6.5 million individuals with a projected increase to 14 million by 2060 [2]. Additionally, between 2000 and 2019, fatalities due to stroke, heart disease, and HIV declined, while the reported deaths attributed to AD increased by over 145%. This increase is primarily attributed to the projected demographic shift toward a higher proportion of elderly individuals in the general population [2, 3]. The population of Americans aged 65 and above is predicted to increase from 58 million in 2021 to 88 million by 2050, which will have a significant impact on healthcare [2, 4, 5]. Moreover, it is important to note the total payments for healthcare, long-term care, and hospice services for individuals aged 65 and above with dementia were estimated at $321 billion in 2022 [2, 4].

AD research has been focused on different aspects including etiology, genetic and environmental risk factors identification, diagnostic methods, and treatment interventions [6–10]. The current drug treatment options for AD are categorized into two groups of FDA-approved pharmacologicals: Cholinesterase inhibitors (such as Donepezil, Rivastigmine, and Galantamine) and NMDA receptor antagonists (namely, Memantine) [11–15]. Donepezil and Memantine are the most commonly prescribed treatments for AD [16–19].

Donepezil is an acetylcholinesterase inhibitor most widely prescribed for treating mild, moderate, and severe Alzheimer’s disease. Clinical trials have suggested Donepezil improves cognitive performance by increasing the levels of acetylcholine in the brain, a neurotransmitter that plays an important role in memory and learning [17]. Whereas, a deficit of acetylcholine is believed to be a significant contributor to the clinical presentation of Alzheimer’s disease. Through FDA tests and other studies, Donepezil has shown a favorable safety profile and sustained tolerability over a long period of time [20, 21]. On the other hand, Memantine is a voltage-dependent, moderate-affinity antagonist of the N-methyl-D-aspartate (NMDA) receptor. It modulates glutamate neurotransmission, which has been implicated in the pathophysiology of AD [18, 19]. Various studies have indicated the effectiveness of Memantine in managing and preventing the behavioral symptoms of AD, particularly in terms of reducing agitation, aggression, and delusions [16, 22–24].

Donepezil and Memantine are generally safe for combined use and, currently, they are also the only AD drugs approved by the FDA to be given in combination. Recent publications have examined the effectiveness of combining Donepezil and Memantine to improve the cognitive function of patients with AD [16, 23, 25]. In this research we examine the impact of the combined use of Memantine and Donepezil on the average number of hospital or emergency department visits per year for Alzheimer’s disease (AD) patients and the corresponding annual treatment cost reduction.

## 2. Data

The main dataset used for these analyses were obtained from the Cerner® Real-World Data (CRWD) database, which is one of the largest multi-center medical databases with accrued data from millions of patients across the United States. It contains detailed information on patient demographics, diagnoses, treatments, procedures, laboratory results, survival, vital signs, and prescription drugs. CRWD has been used to facilitate numerous findings in healthcare research and is considered a valuable resource for patient health outcomes [26–28].

The secondary, hospitalization cost reduction analysis data were obtained from a subset of the IQVIA database, which is a comprehensive healthcare data repository containing detailed information on healthcare providers, patient diagnoses, prescription drugs, insurance claims, and medical devices [29].

### Cerner database

Cerner Real-World Data TM (CRWD) is an electronic de-identified health database that includes information from more than 110 healthcare systems across the United States [26–28]. The database consists of 100,885,181 distinct patients, 1,660,686,928 distinct encounters, 263,436 distinct drug codes, 193,802 unique diagnostic codes (ICD-9 and ICD-10), and billions of measurements overall.

### IQVIA database

The IQVIA database adheres to high privacy protection standards and employs security protocols to maintain patient confidentiality and data integrity [29]. For our analysis, we queried a 25% random sample of the database, spanning 16 years from 2006 to 2022. This sample included 23,390,878 unique patients, 98,177 unique diagnostic codes (ICD-9 and ICD-10), and 2,964,011,729 claims.

### Alzheimer’s disease dataset

**Fig 1:**
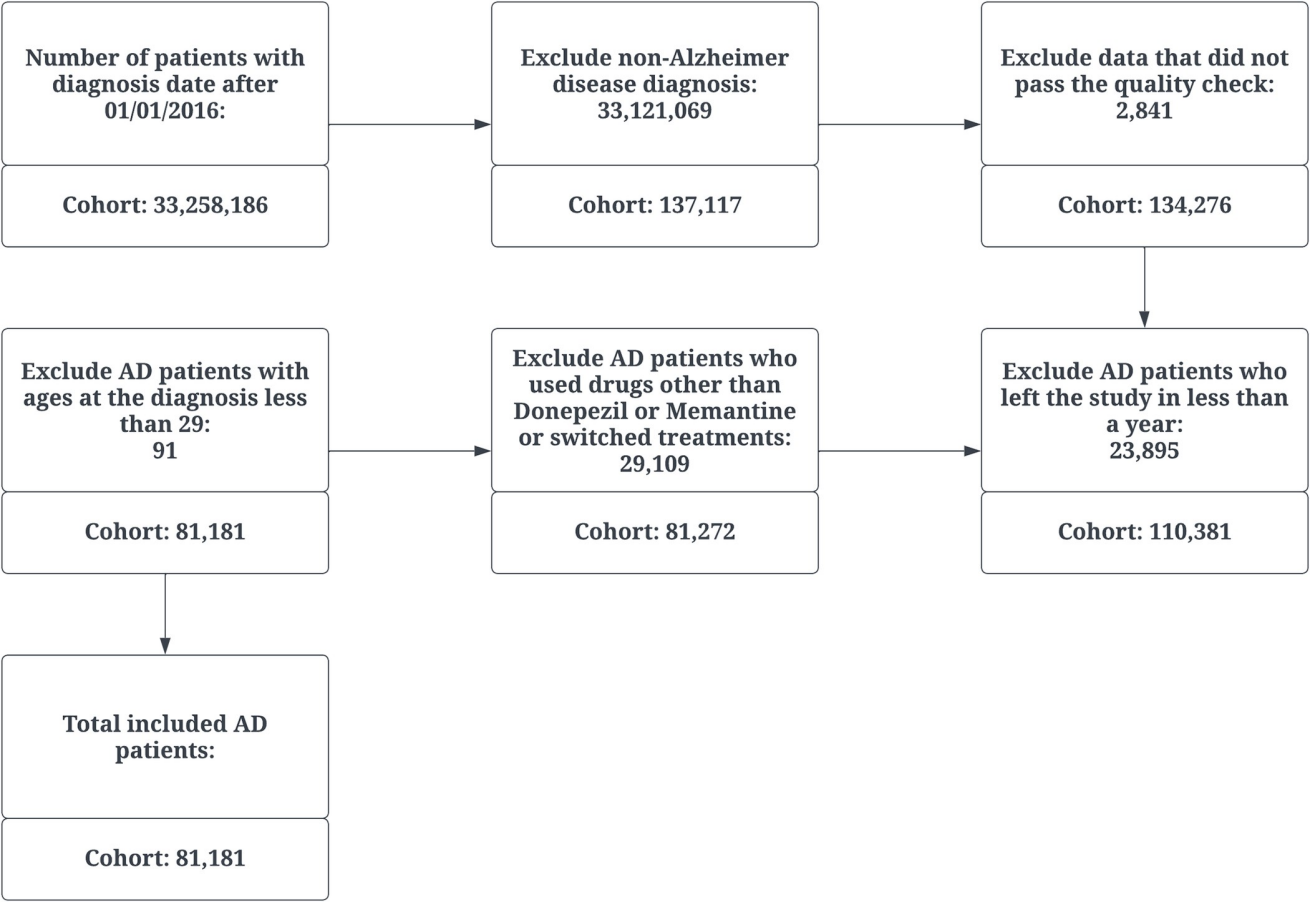
Flowchart of data preprocessing steps.

We identified AD patients using ICD-10 Code G30 and ICD-9 code 331. Initially, there were 137,117 patients diagnosed with AD on or after January 1, 2016. After data quality checks and cleaning, the cohort size was reduced to 134,276 patients. In particular, in the data cleaning steps, we excluded patients with survival durations of less than 0 days, as this indicates a data entry error where their recorded date of death precedes their date of birth. We omitted patients whose service dates were in 2023, as we were unable to monitor them for a full year. We only looked at patients who had service dates from 2016 to 2022. Additionally, we excluded patients with missing service dates or discharge dates as this would hinder our ability to calculate the number of hospital or emergency department visits or the length of stay at each visit. We also removed patients at any time point who were in the hospital for more than 60 days at a time because this could signify a patient waiting for specialized care in hospice service or undergoing surgery. We kept patients that were in our study for at least one year to avoid small sample bias due to insufficient time of follow-up. Further exclusions were made, including patients who used drugs other than Donepezil or Memantine or switched treatments (81,272), and those diagnosed with AD before the age 29, thus a final cohort of 81,181 distinct AD patients. Fig 1 illustrates the sequential data preprocessing steps conducted in the study.

### Outcome variable (The average number of hospital and emergency department visits per year)

Our study was based on data from 81,181 patients who were diagnosed with AD between 2016 and 2022. We calculated the average number of hospital or emergency department visits per year by dividing the number of times the patient admitted to hospital or the emergency department facility during the patient’s time in the study by their total number of years in the study. Table 1 shows the summary statistics for the average number of hospital or emergency department visits per year.

**Table 1:** Summary statistics of the average number of hospital or emergency department visits per year.

	N (%)
**The Average Number of Hospital or Emergency Department Visits per Year**	
0	19,603 (24.15)
(0, 0.25]	11,824 (14.56)
(0.25, 5]	16,666 (20.53)
(0.5, 1]	17,716 (21.82)
1+	15,372 (18.94)
**Number of Hospital Visits**	
**0**	19,603 (24.15)
**1**	28,379 (34.96)
**2**	12,781 (15.74)
**3+**	20,418 (25.15)
**Years In Study**	
**[1,2)**	17,874 (22.02)
**[2,4)**	29,189 (35.96)
**[4,6)**	24,426 (30.09)
**[6, 6+)**	9,692 (11.94)
**Total**	**81,181**

### Alzheimer’s disease medications

We analyzed the effects of two of the most frequently prescribed AD medications: Donepezil and Memantine. Among the 81,181 patients with confirmed AD diagnosis, 39,016 (48.06%) did not receive any medication treatment. Donepezil was the most commonly prescribed medication, with a prevalence of 24,800 (30.55%), followed by Memantine with 9,374 (11.55%). The combined use of Donepezil and Memantine was prescribed to 7,991 (9.84%) patients. Table 2 shows a detailed summary of the medication use of the AD patients in our study.

**Table 2:** Summary statistics of the patients’ Alzheimer’s disease medications use.

Medications	N (%)
**None**	39,016 (48.06)
**Donepezil**	24,800 (30.55)
**Donepezil & Memantine**	7,991 (9.84)
**Memantine**	9,374 (11.55)
**Total**	81,181 (100.00)

We accounted for a selected list of comorbidities in our study, which have the potential to act as confounding factors for the effect of Alzheimer’s disease (AD) treatment on the average number of hospital or emergency department visits. The most prevalent comorbidity was Heart Disease (I3-I5), affecting 39,114 (48.18%) of the patients, followed by Hypertensive Diseases (I10) at 20,050 (24.70%), Cerebral Infarction at 14,777 (18.20%), Diabetes mellitus at 12,641 (15.57%), and Acute Kidney Failure (N17-N19) at 6,853 (8.44%). Table 3 shows a detailed summary of the comorbidities of our AD patient cohort.

**Table 3:** Summary statistics of the distribution of patients’ comorbidities.

Comorbidities	N (%)
**Cerebral Infarction (I60-I69)** *	
**Absence**	66,404 (81.80)
**Presence**	14,777 (18.20)
**Diabetes mellitus (E10-E13)** *	
**Absence**	68,540 (84.43)
**Presence**	12,641 (15.57)
**Overweight And Obesity (E66)** *	
**Absence**	79,164 (97.52)
**Presence**	2,017 (2.48)
**Hypertensive Diseases (I10)** *	
**Absence**	61,131 (75.30)
**Presence**	20,050 (24.70)
**Other Forms of Heart Disease (I3-I5)** *	
**Absence**	42,067 (51.82)
**Presence**	39,114 (48.18)
**Acute Kidney Failure (N17-N19)** *	
**Absence**	74,328 (91.56)
**Presence**	6,853 (8.44)
**Total**	81,181

*Codes in parenthesis are ICD-10 Codes

### Demographic characteristics

To identify patients with Alzheimer’s disease, we used the International Classification of Diseases, Tenth Revision (ICD-10) codes, including G30 (Alzheimer’s disease), G30.0 (Alzheimer’s disease with early onset), G30.1 (Alzheimer’s disease with late onset), G30.8 (Other Alzheimer’s disease), and G30.9 (Alzheimer’s disease, unspecified). Specifically, we selected patients with these codes and a first diagnosis date between January 2016 and December 2022, resulting in a total of 137,112 patients. Out of these, 81,181 patients were either untreated or showed complete treatment adherence to Donepezil and/or Memantine without switching treatments at any point of time.

Among the 81,181 patients with Alzheimer’s disease and complete treatment adherence, 19,603 (24.15%) had no hospital or emergency department visits. The patients who had hospital stays or emergency department visits were further categorized based on their number of hospital or emergency department visits. Among them, 11,824 (14.56%) had between 0 and 0.25 visits per year, 16,666 (20.53%) had between 0.25 and 0.5 visits per year, 17,716 (21.82%) had between 0.5 and 1 visits per year, and 15,372 (18.94%) had more than 1 visits per year.

In the entire study population regarding race/ethnicity, 372 (0.46%) patients self-identified as American Indian or Alaska Native, 1,970 (2.43%) as Asian, 130 (0.16%) as Pacific Islander, 5,730 (7.06%) as Black or African American, 363 (0.45%) as Hispanic or Latino, 64,682 (79.68%) as Caucasian, and 7,934 (9.77%) as belonging to mixed, other, or unknown racial groups. Among the total cohort, the majority of patients (63.04%) were female, 36.89% were male, and 0.07% had unknown or missing sex. The age distribution of the patients was as follows: 25,009 (30.81%) were 85 years or older, 39,256 (48.36%) were between 76–85 years old, 12,843 (15.82%) were between 66–75 years old, 3,885 (4.79%) were between 46–65 years old, and 188 (0.23%) were 45 years old or younger (Table 4). Additionally, 37,806 (46.57%), 29,943 (36.88%), 6,223 (7.67%), and 7,209 (8.88%) were single, married, divorced, and of unknown marital status, respectively. Table 4 shows detailed summary statistics of the patients’ demographic characteristics stratified by treatment and overall.

**Table 4:** Summary statistics of the patients’ demographic characteristics.

	Donepezil	Donepezil/ Memantine	Memantine	No Treatment	Total N (%)
**Age at Diagnosis**					
**< 46**	31 (0.13)	3 (0.04)	9 (0.1)	145 (0.37)	188 (0.23)
**46–65**	1,129 (4.55)	373 (4.67)	362 (3.86)	2,021 (5.18)	3,885 (4.79)
**66–75**	4,325 (17.44)	1,500 (18.77)	1,413 (15.07)	5,605 (14.37)	12,843 (15.82)
**76–85**	12,599 (50.8)	4,106 (51.38)	4,806 (51.27)	17,745 (45.48)	39,256 (48.36)
**85 or older**	6,716 (27.08)	2,009 (25.14)	2,784 (29.7)	13,500 (34.6)	25,009 (30.81)
**Gender**					
**Female**	15,572 (62.79)	4731 (59.2)	6050 (64.54)	24,827 (63.63)	51,180 (63.04)
**Male**	9,207 (37.13)	3,255 (40.73)	3,320 (35.42)	14,164 (36.3)	29,946 (36.89)
**Unknown**	21 (0.08)	5 (0.06)	4 (0.04)	25 (0.06)	55 (0.07)
**Race**					
**African American**	1,844 (7.44)	414 (5.18)	545 (5.81)	2,927 (7.5)	5,730 (7.06)
**American Indian or Alaska Native**	110 (0.44)	18 (0.23)	30 (0.32)	214 (0.55)	372 (0.46)
**Asian**	519 (2.09)	167 (2.09)	270 (2.88)	1,014 (2.6)	1,970 (2.43)
**Caucasian**	20,151 (81.25)	6,661 (83.36)	7,483 (79.83)	30,387 (77.88)	64,682 (79.68)
**Hispanic**	129 (0.52)	36 (0.45)	55 (0.59)	143 (0.37)	363 (0.45)
**Pacific Islander**	37 (0.15)	12 (0.15)	13 (0.14)	68 (0.17)	130 (0.16)
**Unknown**	2,010 (8.1)	683 (8.55)	978 (10.43)	4,263 (10.93)	7,934 (9.77)
**Marital Status**					
**Divorced**	2,034 (8.2)	561 (7.02)	670 (7.15)	2958 (7.58)	6,223 (7.67)
**Married**	9,918 (39.99)	3,395 (42.49)	3,654 (38.98)	12,976 (33.26)	29,943 (36.88)
**Single**	10,870 (43.83)	3,262 (40.82)	4,249 (45.33)	19,425 (49.79)	37,806 (46.57)
**Unknown**	1,978 (7.98)	773 (9.67)	801 (8.54)	3,657 (9.37)	7,209 (8.88)
**Total**	**24,800**	**7,991**	**9,374**	**39,016**	**81,181**

## 3. Methods

One of the classical singly robust causal inference methods is standardization. In essence this is a regression approach that removes confounding by including important variables (identified by expert knowledge external to the data) in a generalized linear model. Additionally, we include a categorical variable for all treatment levels along with interactions between demographics, preexisting conditions, and treatment levels. If the model is correct, we achieve unbiased estimates of the effects of all treatments on the average number of visits for every patient. These subject-specific estimates for all treatments are averaged to obtain population-level effect estimates.

A second classical singly robust causal inference method is IP weighting. This approach is based on creation of a pseudo population that is free of bias. That is achieved by estimating subject specific weights using generalized linear model. If this model is correct, the pseudo population is free from bias and direct estimation of the causal effects of all treatments can be obtained via calculation of simple averages.

The doubly robust estimator combines the standardized and IP weighted estimators in a fashion that produces an unbiased estimator of the casual effects of treatments even if only of the two models is correct [30, 31]. The estimation procedure was adjusted for multiple treatment settings and included the calculation of naïve and Bonferroni-adjusted 95% confidence intervals through nonparametric bootstrapping. The resulting causal effect estimates, and bootstrap samples were then used to derive risk difference and drug-drug interaction estimates, along with their corresponding 95% confidence intervals. More detailed information on the estimation procedure is presented in the following sections.

### Treatments

We only considered treatments that were prescribed to at least 0.5% of the patients. We also excluded patients who switched treatments at any point of time. Therefore, we considered four treatments labeled: 0, 1, 2, and 3, which correspond to no drug use, Memantine monotherapy, Donepezil monotherapy, and the combined use of Memantine and Donepezil, respectively. Among the 81,181 patients included in our study, 39,016 (48.06%) were on treatment 0, 9,374 (11.05%) were on treatment 1, 24,800 (30.55%) were on treatment 2, and 7,991 (9.84%) were on treatment 3.

### Statistical model

Let *Y* be a continuous variable denoting the average number of hospital or emergency department visits per year for a patient and *a* denote the treatment. We use the doubly robust estimator to assess the average causal effects of all four treatments,

$$E(Y^{a=i}),i=0,1,2,3.$$ (1.1)

We treat this setting as a causal inference problem with multiple treatment levels.

Let *L* be a vector that contains all demographic variables, comorbidities, and the squares of the continuous variables. First, we build a multinomial logistic regression model,

$$\log[P(A=i|L)/P(A=0|L)]=\beta_{i}{}^{T}L.$$ (1.2)

From this model we can estimate all probabilities of drug treatment *P*(*A* = *i*|*L*),

$$\hat{P}(A=i|L)=\frac{e^{{\hat{\beta }}_{i}^{T}L}}{1+\sum_{j=1}^{3}e^{{\hat{\beta }}_{j}^{T}L}}={\hat{\pi }}_{i}(L),i=1,2,3,$$ (1.3)

as well as the probability of no treatment,

$$\hat{P}(A=0|L)=\frac{1}{1+\sum_{j=1}^{3}e^{{\hat{\beta }}_{j}^{T}L}}={\hat{\pi }}_{0}(L).$$ (1.4)

Here [*π*_*i*_(*L*)]^−1^, *i* = 0,1,2,3 are the Inverse Probability (IP) weights. Then, the IP weights causal effect estimators of all treatments are given by:

$$E(Y^{a=i})=E(\frac{A_{i}Y}{\pi_{i}(L)}).$$ (1.5)

We can calculate these using the Horwitz-Thompson IP weighted estimators:

$${\hat{E}}_{IP}(Y^{a=i})=\hat{E}(\frac{A_{i}Y}{\pi_{i}(L)})=\frac{1}{n}\sum_{k=1}^{n}\frac{I\{A_{k}=i\}Y_{k}}{{\hat{\pi }}_{i}(L)}.$$ (1.6)

Next, we describe the standardized estimator of the causal effects of all treatments.

Let *b*_*i*_(*L*) denote the average value of *Y* for patients in stratum L that are prescribed treatment *i*,

$$b_{i}(L)=E(Y|A=i,L).$$ (1.7)

We can estimate these values using a linear regression,

$$E(Y|A,L)=\alpha_{1}^{T}L+\alpha_{2}^{T}A+\alpha_{3}^{T}AL,$$ (1.8)

$${\hat{b}}_{i}(L)={\hat{\alpha }}_{1}^{T}L+{\hat{\alpha }}_{2}^{T}A+{\hat{\alpha }}_{3}^{T}AL.$$ (1.9)

Using the double expectation formula, *E*(*Y*^*a = i*^) can be written as,

$$E(Y^{a=i})=E[E(Y|A=i,L)]=E[b_{i}(L)].$$ (1.10)

Therefore, we can calculate the standardized estimators of these using the plug-in g-formula:

$${\hat{E}}_{ST}(Y^{a=i})=\frac{1}{n}\sum_{k=1}^{n}{\hat{b}}_{i}(L).$$ (1.11)

Finally, the doubly robust estimator of *E*(*Y*^*a = i*^) is given by,

$${\hat{E}}_{DR}(Y^{a=i})=\frac{1}{n}\sum_{k=1}^{n}[{\hat{b}}_{i}(L_{k})+\frac{I\{A_{k}=i\}}{{\hat{\pi }}_{i}(L)}(Y_{k}-{\hat{b}}_{i}(L_{k}))].$$ (1.12)

Next, based on these estimates we can estimate the differences of causal effects,

$$RD_{s}=E(Y^{a=3})-E(Y^{a=s}),s=0,1,2.$$ (1.13)

$$\hat{R}D_{s}={\hat{E}}_{DR}(Y^{a=3})-{\hat{E}}_{DR}(Y^{a=s}).$$ (1.14)

### Nonparametric bootstrap 95% confidence intervals for causal effects and differences of causal effects

We calculate 95% confidence intervals for the causal effects using nonparametric bootstrapping. In particular, we create 10,000 datasets *B*_1_,*B*_2_,…,*B*_10000_ of size 81,181 by implementing random sampling of patients with replacement from the original dataset. We applied the doubly robust estimator (1.11) to all *B*_*i*_’*s*, *i* = 0,1,2,3 to obtain 10,000 estimates of the causal effect of *i*^th^ treatment,

$$C_{ij}={\hat{E}}_{DR}^{B_{j}}(Y^{a=i}),j=1,2,\ldots ,10000.$$ (1.15)

We use these values to empirically estimate the distribution of the causal effect estimator ${\hat{E}}_{DR}(Y^{a=i})$, the Bonferroni-adjusted (for four treatments) simultaneous 95% confidence intervals for the causal effects of the four treatments, and the Bonferroni-adjusted (for three treatment differences) 95% confidence intervals for the differences of causal effects between treatment 3 and treatment *s*, *s* = 0,1,2.

### Drug-drug interaction analysis

We investigated the presence of drug-drug interaction (*DDI*) by estimating the following expression,

$$DDI=E(Y^{a=3})-E(Y^{a=2})-E(Y^{a=1})+E(Y^{a=0})$$ (1.16)

We use the doubly robust estimates (1.11) for each counterfactual outcome in (1.16),

$$D\hat{D}I={\hat{E}}_{DR}(Y^{a=3})-{\hat{E}}_{DR}(Y^{a=2})-{\hat{E}}_{DR}(Y^{a=1})+{\hat{E}}_{DR}(Y^{a=0}).$$ (1.17)

Finally, we create the 95% confidence interval for the *DDI* measure using the same nonparametric bootstrapping procedure described above.

## 4. Results

Our results from the treatment analyses show that the estimated causal effects of treatments 0, 1, 2, and 3 on the average number of hospital or emergency department visits per year (and Bonferroni-adjusted 95% CI) were 0.64 (0.63, 0.65), 0.71 (0.69, 0.73), 0.70 (0.68, 0.71), and 0.56 (0.55, 0.58) respectively. Table 5 shows detailed summary statistics from these causal risk effects analyses.

**Table 5:** Casual effect of treatment on the average number of hospital or emergency department visits and 95% confidence intervals.

Causal Effects	Estimates	95% Confidence Intervals	Bonferroni-adjusted 95% Confidence Intervals
***E***(***Y***^***a* = 0**^)	0.64	(0.63, 0.65)	(0.63, 0.65)
***E***(***Y***^***a* = 1**^)	0.71	(0.69, 0.73)	(0.69, 0.73)
***E***(***Y***^***a* = 2**^)	0.70	(0.68, 0.71)	(0.68, 0.71)
*E*(*Y*^*a* = 3^)	0.56	(0.55, 0.58)	(0.55, 0.58)

Our results from the treatment comparison analyses show that the combined use of Donepezil and Memantine significantly decreased the average number of hospital or emergency department visits per year compared to no drug use and Donepezil or Memantine monotherapy. In particular, compared to no drug use, the combined use of Memantine of Donepezil significantly decreased the average number of hospital or emergency department visits per year by 0.078 (13.8%) with a corresponding Bonferroni adjusted 95% CI (0.05, 0.10). Further, compared to the use of Memantine alone, the combined use of Memantine and Donepezil significantly decreased the average number of hospital or emergency department visits per year by 0.14 days (25.5%) with corresponding Bonferroni-adjusted 95% CI (0.11, 0.18). Lastly, compared to the use of Donepezil alone, the combined use of Memantine and Donepezil significantly decreased the average number of hospital or emergency department visits per year by 0.13 (23.4%) with corresponding Bonferroni-adjusted 95% CI (0.11, 0.16). Table 6 shows detailed statistics from these causal risk differences analyses.

**Table 6:** Differences of causal effects of treatment on average number of hospital or emergency department visits and 95% confidence intervals.

Differences of Causal Effects	Estimates	95% Confidence Intervals	Bonferroni-adjusted 95% Confidence Intervals
***E***(***Y***^***a* = 3**^)−***E***(***Y***^***a* = 0**^)	-0.08	(-0.10, -0.06)	(-0.10, -0.05)
***E***(***Y***^***a* = 3**^)−***E***(***Y***^***a* = 1**^)	-0.14	(-0.17, -0.12)	(-0.18, -0.11)
***E***(***Y***^***a* = 3**^)−***E***(***Y***^***a* = 2**^)	-0.13	(-0.15, -0.11)	(-0.16, -0.11)

### Medical cost analysis

The estimated decrease of the average number of hospital or emergency department visits per year apart from the direct health benefits entails a large decrease in the medical cost of treatment for AD patients. Using the IQVIA insurance claims database, we estimated that the average hospitalization cost per AD patient in 2021 was $1,532.62. The database classifies claims into six categories: M (Management), S (Surgical), F (Facility), A (Ancillary), P (Pharmaceutical), and J (a non-clinical code used to net costs associated with a confinement). We used Alzheimer’s disease ICD9 and ICD10 diagnostic codes to query the insurance claims. We dropped the claims with $0 and the P (Pharmaceutical) class claims. The remaining data was used to estimate the average cost per visit per AD patient. As reported by the Alzheimer’s disease Association, there are an estimated 6.5 million patients with Alzheimer’s disease (AD) in the United States. Approximately 2,301,000 (35.4%) are currently on no treatment, 1,475,500 (22.7%) are currently on Donepezil and 552,500 (8.5%) are currently on Memantine. Switching these patients to combined Donepezil and Memantine treatment will decrease the hospital or emergency department visits per year by over 613 thousand and the yearly medical expenses in the U.S. by over 940 million dollars.

### Drug-drug interaction

Our results from the drug-drug interaction analysis show that Memantine and Donepezil have a significant beneficial interaction with respect to the average number of hospital or emergency department visits per year of AD patients. The estimated value of the interaction effect calculated using was -0.20 with a 95% confidence interval (-0.23, -0.17).

## 5. Discussion

The results of our analyses demonstrated that the combined use of Memantine and Donepezil significantly decreased the average number of hospital or emergency department visits per year of AD patients compared to patients that were on no drug treatment and on Memantine and Donepezil monotherapies.

In particular, the adoption of the combined treatment of Memantine and Donepezil in the U.S. for patients that are currently on no drug treatment and on Memantine and Donepezil monotherapies could reduce their average number of hospital or emergency department visits per year by more than 613 thousand with corresponding yearly medical cost reduction of over 940 million dollars. The CDC projected number of AD patients in 2060 is 14 million in the U.S., and the estimated reduction of the average number of hospital or emergency department visits per year will be over 1.3 million with corresponding yearly medical cost reduction of over 2 billion dollars without adjustment for increase of the medical cost in the future or over 4.3 billion dollars if we assume a constant 2% annual increase of medical cost. The Memantine and Donepezil have distinct active components that induce different ways of cognitive improvement. It has been suggested that patients using the combined treatment of the two drugs improve their cognitive performance compared to patients using monotherapy treatments. It could be surmised that the improved cognitive abilities generally improve the quality of life and that subsequently decreases the hospitalization risks and duration.

Our analytic findings provide a valuable suggestion for treatment modification of AD patients that should be approved by and coordinates with the physician.

This study has several potential limitations related to the data availability. Firstly, there was no data on important lifestyle factors, genetic and environmental confounders that may be potential confounders for the effect of treatments on the average number of hospital or emergency department visits per year. Additionally, there was no AD severity variable in the database. Severity could be inferred from the treatment they receive with combined treatment being given to the sickest patients. Thus, the true beneficial effect of the combined treatment is in fact greater than the one we reported. Secondly, there was no information regarding the continuous association of patients with the Cerner network of medical facilities that might entail selection bias due to a differential loss to follow-up. Thirdly, there might be an uncounted placebo effect stemming from the fact that patients on the combined treatment expect to be experience the largest possible benefit among drug treatments. Fourthly, we do not account for insurance types as approximately 70% of the patients had access to Medicare/Medicaid but 26% of them had not reported their source of pay. Therefore, subsequent studies are needed to address these limitations and to advance our understanding of the advantages of the combined use of Memantine and Donepezil for Alzheimer’s disease patients.

## Abbreviations

AD: Alzheimer’s disease
CDC: Centers for Disease Control and Prevention
ChEI: Choline Esterase Inhibitors
CI: Confidence Interval
CRWD: Cerner® Real World Data
DDI: drug-drug interaction
FDA: Food and Drug Administration
ICD: International Classification of Diseases
IP: Inverse Probability
IPW: Inverse Probability Weights
NMDA: N-methyl-D-aspartate
